# Integration of semi-*in vivo* assays and multi-omics data reveals the effect of galloylated catechins on self-pollen tube inhibition in *Camellia oleifera*

**DOI:** 10.1093/hr/uhac248

**Published:** 2022-11-10

**Authors:** Yihong Chang, Wenfang Gong, Jinming Xu, Han Gong, Qiling Song, Shixin Xiao, Deyi Yuan

**Affiliations:** Key Laboratory of Cultivation and Protection for Non-Wood Forest Trees of the Ministry of Education and Key Laboratory of Non-Wood Forest Products of the Forestry Ministry, Central South University of Forestry and Technology, Changsha, Hunan 410004, China; Key Laboratory of Cultivation and Protection for Non-Wood Forest Trees of the Ministry of Education and Key Laboratory of Non-Wood Forest Products of the Forestry Ministry, Central South University of Forestry and Technology, Changsha, Hunan 410004, China; Key Laboratory of Cultivation and Protection for Non-Wood Forest Trees of the Ministry of Education and Key Laboratory of Non-Wood Forest Products of the Forestry Ministry, Central South University of Forestry and Technology, Changsha, Hunan 410004, China; Key Laboratory of Cultivation and Protection for Non-Wood Forest Trees of the Ministry of Education and Key Laboratory of Non-Wood Forest Products of the Forestry Ministry, Central South University of Forestry and Technology, Changsha, Hunan 410004, China; Key Laboratory of Cultivation and Protection for Non-Wood Forest Trees of the Ministry of Education and Key Laboratory of Non-Wood Forest Products of the Forestry Ministry, Central South University of Forestry and Technology, Changsha, Hunan 410004, China; Key Laboratory of Cultivation and Protection for Non-Wood Forest Trees of the Ministry of Education and Key Laboratory of Non-Wood Forest Products of the Forestry Ministry, Central South University of Forestry and Technology, Changsha, Hunan 410004, China; Key Laboratory of Cultivation and Protection for Non-Wood Forest Trees of the Ministry of Education and Key Laboratory of Non-Wood Forest Products of the Forestry Ministry, Central South University of Forestry and Technology, Changsha, Hunan 410004, China

## Abstract

*Camellia* oil extracted from the seeds of *Camellia oleifera* Abel. is a popular and high-quality edible oil, but its yield is limited by seed setting, which is mainly caused by self-incompatibility (SI). One of the obvious biological features of SI plants is the inhibition of self-pollen tubes; however, the underlying mechanism of this inhibition in *C. oleifera* is poorly understood. In this study, we constructed a semi-*in vivo* pollen tube growth test (SIV-PGT) system that can screen for substances that inhibit self-pollen tubes without interference from the genetic background. Combined with multi-omics analysis, the results revealed the important role of galloylated catechins in self-pollen tube inhibition, and a possible molecular regulatory network mediated by *UDP-glycosyltransferase* (*UGT*) and *serine carboxypeptidase-like* (*SCPL*) was proposed. In summary, galloylation of catechins and high levels of galloylated catechins are specifically involved in pollen tube inhibition under self-pollination rather than cross-pollination, which provides a new understanding of SI in *C. oleifera*. These results will contribute to sexual reproduction research on *C. oleifera* and provide theoretical support for improving *Camellia* oil yield in production.

## Introduction

Apart from using the leaves for tea and the flowers for ornamental purposes, the extraction of edible oil from the seeds is another major use of plants of the genus *Camellia*. In particular, the species *Camellia oleifera* Abel. is widely grown because of the high-quality edible oil obtained from its seeds, which contain abundant amounts of unsaturated fatty acids (90%), oleic acid (80%), and several other nutritious substances [1]. With its various health care functions, *Camellia* oil is not only used in the food and pharmaceutical industries, but is also widely used in the cosmetics industry [2]. To preserve fertile farmland for growing major food crops, planting *C. oleifera* on hillsides with a gradient of 5°~25°, where the planting of other food crops is unsuitable, is one of China’s food security strategies in the face of limited farmland. However, an important factor limiting the yield of *Camellia* oil is its self-incompatibility (SI).

SI is a reproductive barrier that is capable of generating and maintaining genetic diversity within a species. The main biological characteristics of SI plants are self- and non-self-recognition processes between the pollen and pistil, followed by selective inhibition of the self-pollen tube. There are two different forms of SI, sporophytic self-incompatibility (SSI) and gametophytic self-incompatibility (GSI), based on the genetic behavior and phenotype of the self-pollen tube inhibition [3]. The mechanisms of self-pollen tube inhibition leading to SI occurrence vary in different plants. In the Brassicaceae family, the inhibition of self-pollen tubes begins with the identification of self-pollen grains and stigmas in SSI *Brassica*, *Arabidopsis*, and *Capsella* species. At this stage, the specific recognition of two *S*-factors, *S*-locus cysteine-rich/*S*-locus protein 11 (SCR/SP11) and *S*-locus receptor kinases (SRKs), induces papillary cell signaling and prevents pollen hydration together with other non-*S*-factors, such as *M*-locus protein kinase (MLPK), Arm repeat-containing proteins (ARC1), and Exo70A1 [4–8]. In GSI plants such as Solanaceae, Plantaginaceae, Rosaceae, and *Citrus* (Rutaceae), self-pollen tube growth is hindered by increases in the accumulation of tip-localized reactive oxygen species (ROS), nuclear DNA degradation, and actin cytoskeleton depolymerization in pollen tubes because the *S*-locus F-box (SLF) protein is not resistant to the toxicity of the S-RNases secreted in the style, which are encoded by the same *S*-haplotype [9–13]. In *Papaver rhoeas*, with the specific recognition of *P. rhoeas pollen S* (*PrpS*) and *P. rhoeas stigma S* (*PrsS*), self-recognition rapidly induces a cascade of physiological responses in the pollen tubes, such as an increase in cytosolic Ca^2+^ followed by downstream changes in ROS, nitric oxide (NO) and pH, as well as depolymerization and reorganization of the actin cytoskeleton, culminating in programmed cell death before stigma penetration [14–16].

Unlike the growth characteristics of pollen tubes belonging to the SSI and GSI forms, in which the pollen tubes either stop growing on the stigma or at various locations of the style, the self-pollinating pollen tubes of *C. oleifera* can grow longer than 10 mm and are arrested near the ovary [17, 18]. However, the mechanism underlying the inhibition of self-pollen tubes in *C. oleifera* has not yet been reported. Researchers have tried to use omics to explore the factors controlling the inhibition of self-pollen tubes, and flavonoid metabolism has been found to have a positive effect on the SI of the genus *Camellia* based on the enrichment analysis [19, 20]. Different types of flavonoids have been shown to regulate pollen tube growth and fertility in various plants [21–24]. Catechins, one of the major flavonoid compounds, have been detected at high levels and polymorphisms in diverse tissues of the genus *Camellia* [25–27]. The main scientific questions that remain are whether catechins contribute to the SI of the genus *Camellia* and what detection methods should be applied other than single omics, which have potential reliability risks.

The pollen tube semi-*in vivo* growth assay enables the successful identification of pollen tube growth attractants from synergid cells [28, 29]. Similarly, inhibitors of pollen tube growth can be identified when SI occurs in *C. oleifera* if there are female-derived inhibitors that contribute to self-pollen tube inhibition. Here, we constructed a semi-*in vivo* pollen tube growth test (SIV-PGT) system with two relatively homogeneous environments for the survival of cross-pollinated (CP) and self-pollinated (SP) pollen tubes protruding from style incisions. High-throughput sequencing and catechin treatment tests were then used to uncover the metabolites and proteins in the environments that inhibit the growth of SP pollen tubes and to explore the related underlying mechanisms. Screening substances related to self-pollen tube inhibition provides a new view of the SI of *C. oleifera* and also helps to promote research on sexual reproduction in other plants.

## Results

### A newly constructed SIV-PGT system demonstrated the important role of the ovary in the self-pollen tube inhibition of *C. oleifera*

**Figure 1:**
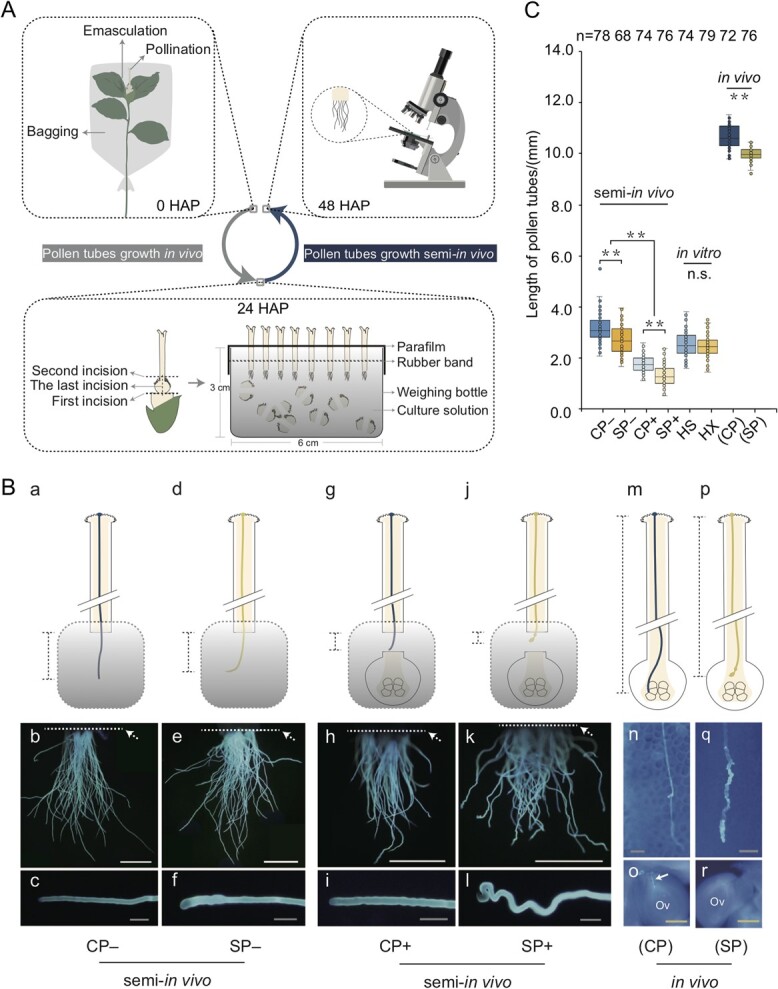
Semi-*in vivo* pollen tube growth test (SIV-PGT) and the inhibition of self-pollen tubes growing *in vivo* or semi-*in vivo*. **A** The operation flow of the SIV-PGT system. Note that only one of the three pollination combinations (unpollinated/UP: ‘HX’, cross-pollination/CP: ‘HX’ × ‘HS’, self-pollination/SP: ‘HX’ × ‘HX’) is illustrated here as an example, with the remaining two operating in the same way. **B** The phenotypes of the CP and SP pollen tubes under both *in vivo* and semi-*in vivo* conditions. CP–/SP– indicates no ovaries in the SIV-PGT device, CP+/SP+ indicates that ovaries were included in the SIV-PGT device, and (CP)/(SP) indicates pollen tubes growing *in vivo*. The dashed box indicates the culture vessel, and the gray gradient indicates the culture solution. Pollen tubes at 48 hours after pollination (HAP) that were photographed after aniline blue staining were used to characterize differences between CP and SP, and between *in vivo* and semi-*in vivo* conditions. Ov indicates ovules. Solid arrows indicate pollen tubes penetrating the ovule; dashed arrows indicate style incisions. The dashed line segment indicates the portion of the pollen tubes used for length measurements. White scale bars, 1 mm; gray scale bars, 50 μm; and yellow scale bars, 200 μm. **C** The differences in the length of ‘HS’ and ‘HX’ pollen tubes grown for 48 h semi-*in vivo*, *in vitro*, and *in vivo*. The pollen tube length semi-*in vivo* only counts the part protruding from the style incision, and the part that grows in the style is not included. n = denotes the number of pollen tubes. ^**^ = values that were determined by *t*-test with a significant difference (*P* < 0.01) between the two groups. n.s. = no significant difference.

By combining the pollen tube orientation assay and semi-*in vivo* pollen tube-secretome assay, we constructed a new semi-*in vivo* pollen tube growth test (SIV-PGT) system. As shown in Fig. 1A, the procedure for constructing this system began with strict pollination. Pollination combinations included cross-pollination (CP) of ‘Huaxin’ (HX) × ‘Huashuo’ (HS) and self-pollination (SP) of ‘HX’ × ‘HX’, and unpollinated (UP) ‘HX’ was used as a control. After 24 h of growth of the germinating pollen grains *in vivo*, the whole pistils were taken off and assembled into the SIV-PGT device, and the pollen tubes were grown semi-*in vivo* for an additional 24 h. Phenotypes of the pollen tubes protruding from the style incision were then observed.

With support from the SIV-PGT system, we first attempted to simulate the SI and cross-compatibility of *C. oleifera* under semi-*in vivo* conditions. *In vivo*, CP *C. oleifera* pollen tubes had normal morphology and could penetrate the micropyle to complete the life course (Fig. 1B – m, n, and o), and the pollen tube length could reach 10 mm (Fig. 1C – *in vivo*). In contrast, SP pollen tubes showed inhibited phenotypes such as bifurcation and swelling near the ovary, and they stopped growing before penetrating the micropyle (Fig. 1B – p, q, and r). Additionally, their length was significantly shorter than that of CP pollen tubes (Fig. 1C – *in vivo*). The significant differences in morphology and length between CP and SP pollen tubes were not due to the pollen tubes themselves, as neither morphology nor length was significantly different when the ‘HX’ and ‘HS’ pollen tubes were grown in the same environment *in vitro* (Fig. S1 (see online supplementary material) and Fig. 1C – *in vitro*). Here we used pollen tube morphology and length to indicate pollination compatibility. Under semi-*in vivo* conditions, the SP pollen tubes (Fig. 1B – j, k, and l) appeared twisted compared to those of CP (Fig. 1B – g, h, and i). Additionally, their length protruding from the style incision at the time of growth arrest was also significantly shorter (Fig. 1C). These two typical characteristics of SI were similar to those observed *in vivo*, indicating that the SIV-PGT system can effectively reflect self-pollen tube inhibition in *C. oleifera*.

The important role of the ovary in self-pollen tube inhibition in *C. oleifera* can be clarified by considering whether the ovary was added to the SIV-PGT device. As shown in Fig. 1B – d, e, and f, the SP pollen tubes showed slight swelling in the SIV-PGT system without ovaries, whereas CP pollen tubes behaved normally in the same environment (Fig. 1B – a, b, and c). After adding ovaries to the SIV-PGT system, the CP pollen tubes continued to grow normally (Fig. 1B – g, h, and i), whereas SP pollen tube growth was significantly inhibited (Fig. 1B – j, k, and l). In the absence of ovaries (CP– and SP–) in the SIV-PGT system, the SP pollen tube length was 85.44% of that of the CP pollen tube length (Fig. 1C). After the addition of the ovary (CP+ and SP+), the difference between the pollen tube lengths of CP and SP increased (Fig. 1C, the pollen tube length of SP was 73.35% that of CP). This suggests that the addition of the ovary exacerbated the inhibition of the self-pollen tubes in the SIV-PGT system.

### Metabolite and protein abundance profiles of the culture solution in the SIV-PGT system during *C. oleifera* self-pollen tube inhibition

**Figure 2:**
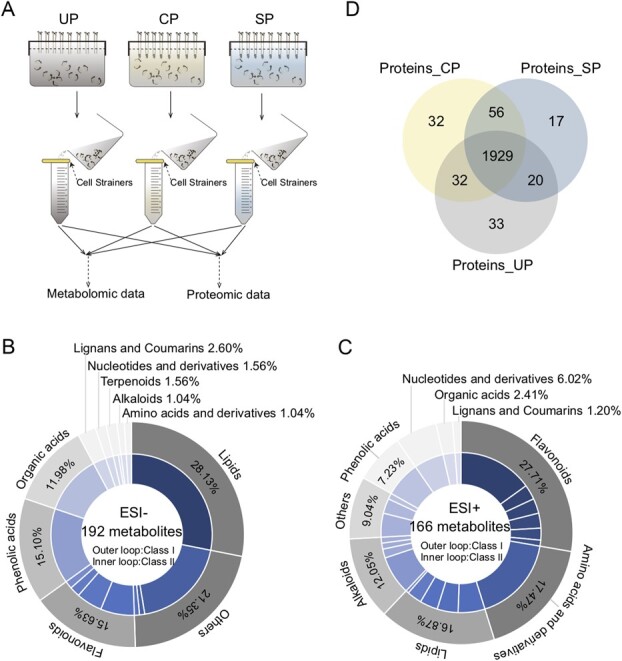
Metabolite and protein abundance profiles of the culture solution in the SIV-PGT system. **A** The workflow for metabolomic and proteomic data collection to determine the pattern of change in the composition of the UP, CP, and SP culture solution substances in the SIV-PGT system at 48 HAP. **B** and **C**, respectively, show the classification and number of metabolites detected in all culture solution samples under ESI− and ESI+ conditions. The colors of the sectors indicate the different types of metabolites, and their area indicates the quantity. The inner loop shows the type and quantity sub-categories of the metabolites in the outer loop (for details, refer to Table S1 and Table S2, see online supplementary material). **D** Venn diagram of the proteins detected in all culture solution samples for UP, CP, and SP in the SIV-PGT system.

Similar to the *in vivo* results, the SP pollen tubes were strongly inhibited in the SIV-PGT medium that included ovaries. The CP and SP pollen tubes exhibited different compatibility, indicating that the substance composition of the original simple and uniform culture solution in the two SIV-PGT systems changed after the pollen tubes protruded from the style incision. In other words, the distinct composition of the culture solution contributed to pollination compatibility. To explore the discriminations among these substances, we separately collected the UP/CP/SP culture solution from three ovary-including SIV-PGT systems at 48 HAP to examine the abundance profiles of metabolites and proteins (Fig. 2A; Fig. S2A, see online supplementary material).

The high consistency of the retention times and peak intensities ensured reliable data for the 358 metabolites [192 negative ion electrospray ionization (ESI−) and 166 positive ion electrospray ionization (ESI +)] detected in at least one biological replicate from UP/CP/SP (Fig. S2B–C and Table S1–S2, see online supplementary material). The results suggested that all 358 detected metabolites were commonly distributed, either in the presence or absence of pollen tubes, or when pollen tube growth was inhibited. All metabolites were classified into 10 and 9 groups for the ESI− and ESI+ models, respectively, with lipids and flavonoids accounting for the highest proportions overall at 22.91% (28.13% ESI− and 16.87% ESI+) and 21.23% (15.63% ESI− and 27.71% ESI+), respectively (Fig. 2B–C).

A total of 5698 peptides were detected in all culture solution samples, with a distribution of lengths consistent with the quality control requirements (Fig. S2D–E, see online supplementary material). Subsequently, 2119 proteins were identified (Table S3, see online supplementary material). Similar to the distribution pattern of metabolites in the three SIV-PGT systems, the majority of identified proteins (1929, 91.03%) also showed a common distribution in UP/CP/SP; however, 8.97% of the proteins showed differential distribution, and the number of SP-specific proteins was lower than that of UP- and CP-specific proteins (17 versus 33 and 32) (Figure 2D). The protein size distribution showed a high frequency of identified proteins ≤60 kDa (Fig. S2F, see online supplementary material). Protein classification according to annotations in the GO database highlighted the terms ‘cellular process’, ‘metabolic process’, ‘cell’, ‘intracellular’, ‘catalytic activity’, and ‘binding’ (Fig. S2G, see online supplementary material). In summary, the proteins in the culture solution of the SIV-PGT system were mainly small and moderately secreted proteins with catalytic, binding, and metabolic functions.

### High levels of galloylated catechins contributed to the inhibition of self-pollen tubes in *C. oleifera*

**Figure 3:**
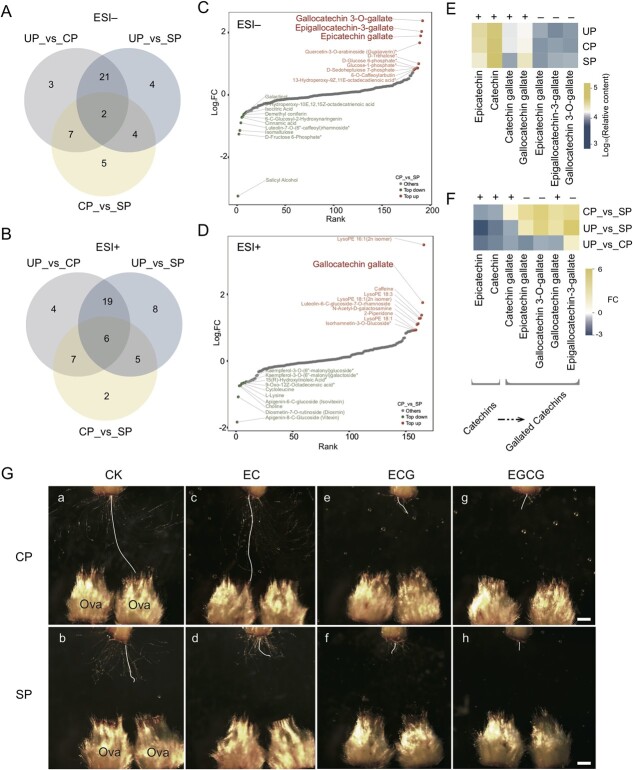
Analysis of differentially abundant metabolites (DAMs) and the important role of galloylated catechins in the inhibition of self-pollen tubes in *Camellia oleifera*. **A**–**B** Venn diagrams of DAMs of UP_vs_CP, UP_vs_SP, and CP_vs_SP. Metabolite analysis was performed using ESI− (**A**) or ESI+ (**B**). **C**–**D** The fold change distribution in CP_vs_SP for all metabolites, among which the top 10 are assigned colors, with red indicating upregulation and green indicating downregulation. The analysis was performed using ESI− (**C**) or ESI+ (**D**). **E** The relative contents of all non-galloylated and galloylated catechins detected from all samples. **F** The difference between all non-galloylated and galloylated catechins in UP_vs_CP, UP_vs_SP, and CP_vs_SP. **G** Growth of pollen tubes on medium containing different catechins based on the SIV-PGT system. Ova refers to an ovary cut in half lengthwise. Scale bars, 1 mm.

There were 48 signals (23 ESI− and 25 ESI+) co-existing in the differentially abundant metabolites (DAMs) of UP_vs_CP and UP_vs_SP (Fig. 3A–B), indicating that the metabolites were essential for the maintenance of CP and SP pollen tube growth in the SIV-PGT system. In particular, the abundances of two ESI− (6-C-glucosyl-2-hydroxynaringenin and epigallocatechin-3-gallate) and six ESI+ (L-glutamine, L-lysine, caffeic aldehyde, vitexin, lysoPE 16:1, and lysoPE 18:3) metabolites in CP_vs_SP significantly differed, which might have contributed to the differences in CP and SP pollen tube phenotypes. In total, five ESI− and two ESI+ metabolites were present only in the DAMs of CP_vs_SP. They showed opposite abundance changes, accompanying the phenotypic differences between CP and SP pollen tubes; for example, the abundance of epicatechin gallate increased with the inhibition of self-pollen tubes in SP but decreased with pollen tube growth in CP (Fig. 3A–B; Table S1, see online supplementary material).

Thirty-eight DAMs (18 ESI− and 20 ESI+) present in CP_vs_SP may have directly contributed to the differences between CP and SP pollen tubes in the SIV-PGT system (Fig. 3A–B). Biosynthesis of secondary metabolites and flavonoids was typical of the pathway enrichment of the 38 DAMs (Fig. S3A–B, see online supplementary material), and in these two pathways, gallocatechin 3-O-gallate, epigallocatechin-3-gallate, epicatechin gallate (ESI−), and gallocatechin gallate (ESI+) had the highest fold changes (3.71–5.19 folds) of increasing abundance in SP compared with that in CP (Fig. 3C–D). This suggests that the elevated galloylated catechins in the SIV-PGT system were closely related to the inhibitory phenotype of the SP pollen tubes.

To clarify the distribution patterns of catechins in UP/CP/SP, all seven monomers of catechins detected in each sample were screened. Overall, non-galloylated catechins showed higher levels than galloylated catechins in all the samples. In addition, non-galloylated catechins made up less than half of UP when pollen tubes appeared in the culture solution in the SIV-PGT systems regardless of CP/SP, suggesting the important role of non-galloylated catechins in pollen tube growth (Fig. 3E–F). Compared to UP, the changes in the abundance of galloylated catechins in the culture solutions of CP and SP differed. Specifically, the abundance of epicatechin gallate, gallocatechin 3-O-gallate, and gallocatechin gallate increased in SP but decreased in CP, while the abundance of epigallocatechin-3-gallate was elevated in both CP and SP, but the elevation was greater in SP. Catechin gallate was reduced in both CP and SP, but the reduction was greater in CP. Therefore, with the inhibition of self-pollen tubes, the abundance of galloylated catechins in the SP culture solution was 1.08 to 5.19 times higher than that of CP (Fig. 3E–F).

To verify the effect of catechins on the growth of *C. oleifera* pollen tubes, pollen tubes grown *in vitro* were treated with different concentrations of non-galloylated catechins and galloylated catechins. The results showed that a certain range of high concentrations of galloylated catechins significantly inhibited the growth of pollen tubes (Table S4 and Fig. S3C, see online supplementary material). To further verify the effect of catechins on CP and SP pollen tubes, 100 μg·mL^−1^ of non-galloylated catechins (epicatechin, EC) and galloylated catechins (epicatechin gallate, ECG; epigallocatechin gallate, EGCG) were added to the culture medium. Semi-*in vivo*, the CP pollen tubes were significantly inhibited by the galloylated catechins (Fig. 3G – e and g), whereas they were not significantly affected by the non-galloylated catechins (Fig. 3G – c). The SP pollen tubes were also significantly inhibited by the galloylated catechins (Fig. 3G – f and h), but the non-galloylated catechins slightly exacerbated the inhibition of self-pollen tubes compared to the control (Fig. 3G – b and d). Therefore, galloylated catechins play an important role in the inhibition of self-pollen tubes in *C. oleifera*.

### Protein profiles closely related to the abundance of different catechin monomers

**Figure 4:**
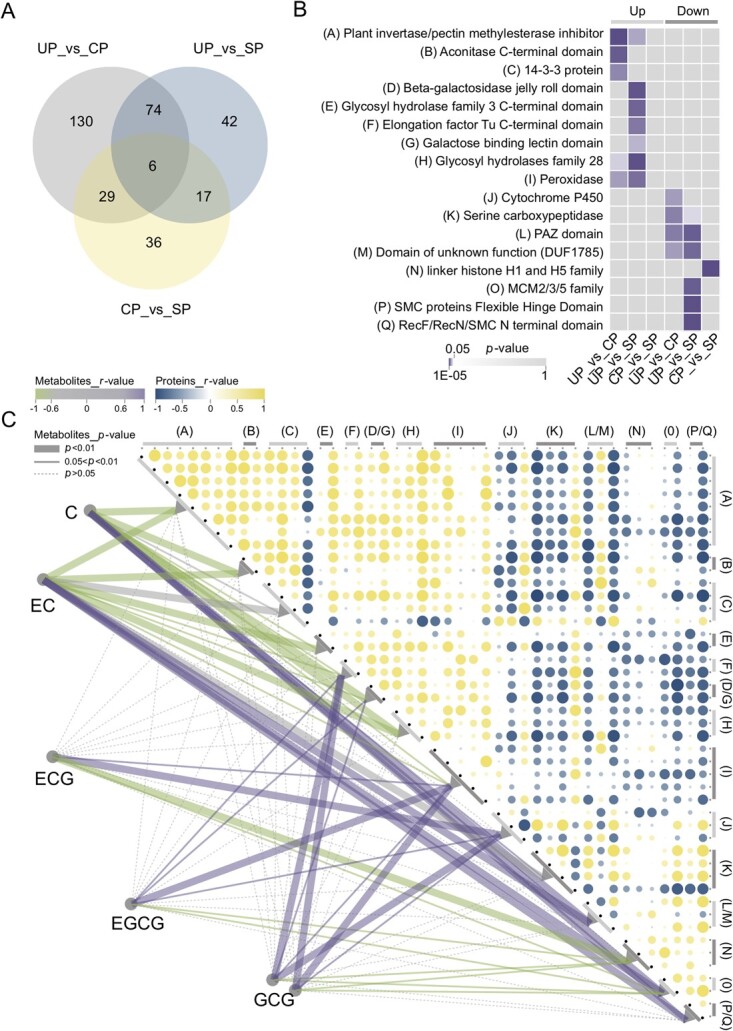
Analysis of differentially abundant proteins (DAPs) and their correlation with different catechins. **A** Venn diagrams of 334 DAPs (UP_vs_CP, UP_vs_SP, CP_vs_SP). **B** Domain enrichment-based clustering of 334 DAPs. **C** Interrelationship of 45 DAPs from domain enrichment-based clustering and their respective correlations with different catechins. The *P*-value of the correlation between catechin gallate and the 45 proteins does not meet the statistical requirements (*P* < 0.05 and |*r*| ≥ 0.8), so it is not shown in the figure.

As shown in Fig. 4A, 334 differentially abundant proteins (DAPs) were identified in UP_vs_CP, UP_vs_SP, and CP_vs_SP. A total of 80 proteins showed significant changes in abundance compared to UP when CP and SP pollen tubes were grown in the culture solution in the SIV-PGT system, suggesting their roles in pollen tube growth. Among them, the abundance of six proteins differed significantly between CP and SP (Fig. 4A), suggesting that they might be involved in the morphological differences between CP and SP pollen tubes.

In total, 36 DAPs that differed significantly in abundance only in CP_vs_SP exhibited diametrically opposite changes in abundance, accompanied by normal growth of CP pollen tubes and inhibition of self-pollen tubes (Fig. 4A). KEGG classification and enrichment of these 36 proteins showed that they were mainly involved in the metabolism of amino sugars, nucleotide sugars, flavones, and flavonols (top two enriched pathways, Fig. S4A–B, see online supplementary material).

Seventeen protein families were obtained by domain enrichment-based clustering of all 334 DAPs, and these families contained 45 members (Fig. 4B; Table S5, see online supplementary material). Significant differences in the abundance of these 45 proteins, which included members of the plant invertase/pectin methylesterase inhibitor family and the glycosyl hydrolase family (18% and 16%, respectively), contributed to the different phenotypes of the CP and SP pollen tubes in the SIV-PGT system. Because catechins were found to be closely related to the compatibility of pollen tubes, we explored the connection between DAPs and different abundances of catechins. Correlation analysis showed that all 45 proteins were correlated with the abundance of different catechin monomers (Fig. 4C). Overall, non-galloylated and galloylated catechins were significantly associated with protein families 15 and 7, respectively. The protein families of the elongation factor Tu C-terminal domain, beta-galactosidase jelly roll domain, galactose-binding lectin domain, peroxidase, and MCM2/3/5 were all significantly associated with non-galloylated and galloylated catechins, but the trend of correlations was reversed. Cytochrome P450 and linker histone H1 and H5 protein families unrelated to non-galloylated catechins were significantly positively and negatively correlated with galloylated catechins, respectively.

Serine carboxypeptidase-like (SCPL) with a conserved domain serine carboxypeptidase is an important enzyme in the catechin biosynthesis pathway [30, 31]. A total of 13 SCPL proteins were identified, among which different members had different correlations with non-galloylated catechins, but none had a significant correlation with galloylated catechins. Additionally, SCPL was extensively correlated with other domain-enriched proteins (Fig. 4C). These results suggest that the action of SCPL and its multidimensional connection with other proteins may account for the differences in catechin abundance.

### *UGT* and *SCPL* are involved in the mechanism underlying the contribution of galloylated catechins to SI in *C. oleifera*

**Figure 5:**
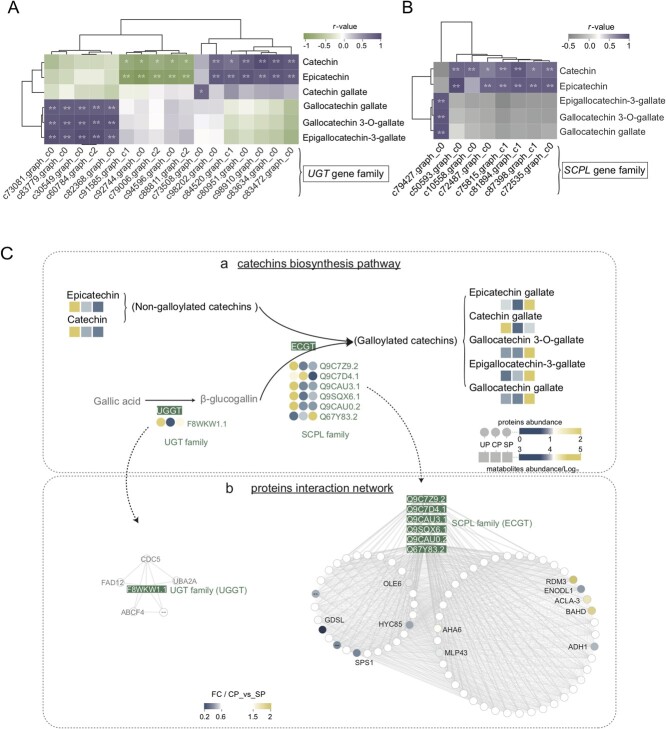
Combined multi-omics analysis and the key pathway of galloylated catechin biosynthesis. **A** and **B** Heat map of the correlation between *UGT* and *SCPL* gene expression levels and the relative contents of different catechins, respectively. The map only shows the results with the thresholds of *P* < 0.05 and |*r* | ≥ 0.8. ^**^P < 0.01; ^*^0.01 < *P* < 0.05. **C** Pathway diagram for the biosynthesis of galloylated catechins from non-galloylated catechins. It shows the relative content of metabolites and proteins as heat maps, as well as a network of interactions between UGGT (UGT family) and ECGT (SCPL family) proteins and other identified proteins. The proteins that interacted with SCPL proteins are displayed with degree values, small circles (degree ≥150), and large circles (130 < degree <150). The names of the putative ECGT and UGGT proteins are represented by the best match of the sequence blast on NCBI, and the names of the other proteins are based on the annotation of the proteomic data.

The *UDP-glycosyltransferase* (*UGT*) and *SCPL* gene families play important roles in the biosynthesis of galloylated catechins using non-galloylated catechins as substrates [31, 32]. The proteomic results showed that more than 83% of the members of the UGT/SCPL protein family were more abundant in SP than in CP (Fig. 5C – a). Moreover, the UP/CP/SP pistils at 48 HAP were used for transcriptome sequencing, and 135 and 49 members belonging to the *UGT* and *SCPL* families, respectively, were identified (Tables S6 and S7, see online supplementary material). Correlation analysis revealed that six *UGT* and one *SCPL* family genes were significantly positively correlated with galloylated catechin synthesis (Fig. 5A–B, |*r* | ≥ 0.8, *P* < 0.05). The qRT-PCR results further verified that the expression of these genes was higher in SP than in CP (Fig. S5 and Tables S9, see online supplementary material). In addition, the activities of the UGT and SCP protein families in SP pistils were significantly higher than those in CP (Fig. S6, see online supplementary material).

Seven UGT (UGGT) and 13 SCPL (ECGT) family members were identified. To understand the mode of action of these two families of protein, we constructed protein interaction networks based on all 1137 quantifiable proteins (Fig. 5C – b). Regarding the first key component of galloylated catechin biosynthesis, only one UGGT (*|r |* ≥ 0.8, *P* < 0.05) was co-expressed with proteins such as ABC transporter F family member 4 (ABCF4) and UBP1-associated protein 2A (UBA2A), which are related to pollen tube growth. Other pollen tube growth-related proteins such as BAHD acyltransferase, ATPase 6-plasma membrane-type (AHA6), GDSL esterase/lipase, major pollen allergen Ole e 6 (OLE6), ADH1 alcohol dehydrogenase 1 (ADH1), and early nodulin-like protein 1 (ENODL1) were co-expressed with six ECGT proteins (*|r |* ≥ 0.8, *P* < 0.05), which are the second key factor in galloylated catechin biosynthesis. Furthermore, the abundance of these proteins was significantly different in CP_vs_SP, which may have contributed to the differential distribution of galloylated catechins, together with the six ECGT proteins.

## Discussion

### A newly constructed SIV-PGT method precisely screens substances that inhibit self-pollen tubes in *C. oleifera*

*Camellia* oil from *C. oleifera* seeds is a high-quality edible oil, but the SI of *C. oleifera* limits its yield [2, 27]. Given that self-pollen tube inhibition is the key reason for SI, researchers first tried to use anatomy and cytology to explain the temporal, spatial, and cellular characteristics of *C. oleifera* when this inhibition occurred. This led to the theory of late-acting self-incompatibility of *C. oleifera*, which is different from GSI and SSI [17, 18]. Then, scientists used a high-throughput sequencing approach to identify the factors that regulate self-pollen tube inhibition. Unpollinated pistils and pistils subjected to 48 HAP cross- and self-pollination were the main materials used to construct libraries of mRNAs, proteins, and metabolites. However, the results obtained using these materials are diametrically inconsistent [19, 20, 33–35]. The reason for these inconsistencies might be differences in the genetic background of the pollen tubes inside the pistils used for sequencing. The low biomass and sensitivity of pollen tubes are precise considering the loud noise from the pistil. Thus, the rough screening of different factors from experimental materials with genetic background differences that may sufficiently influence single-cell pollen tubes may result in unrepeatable results from multiple trials.

SIV-PGT is a new method based on the pollen tube orientation assay [28, 29, 36–38] and the semi-*in vivo* pollen tube-secretome assay [39, 40]. In this study, the SIV-PGT system provided a simple and uniform living environment for CP and SP pollen tubes. Along with significant differences in pollen tube phenotypes between CP and SP, the metabolites and proteins identified from the corresponding culture environments specifically indicate the SI and cross-compatibility of *C. oleifera* without interference from background noise. Moreover, the identified factors that are specifically related to pollen tubes growing*in vivo* [41] (cytochrome P450, serine carboxypeptidase) also appeared in the DAP enrichment results of this study, which shows that these results can accurately reflect the factors specifically related to pollen tube growth and can also greatly reflect the differences between CP and SP pollen tubes. The simulation of self-pollen tube inhibition *in vitro* is an important technical means to study the mechanism of SI in perennial woody plants. There are many breakthroughs based on the *S-RNase in vitro* simulation system for apple [42–44] and pear [45, 46]. Therefore, without knowing the SI determinants (such as *S-RNase* in pears and apples), the incompatibility factors screened with the SIV-PGT system will be introduced into the medium in a semi-*in vivo* manner, and the response of the inhibitory phenotype of self-pollen tubes will be observed after the change in the medium composition.

The drawback of the SIV-PGT system is that it can only efficiently detect secretable substances that regulate pollen tube growth, such as metabolites and proteins. However, based on the SIV-PGT system, we found that these secreted substances were sufficient to induce strong self-pollen tube inhibition, which suggested that the secreted substances may be the main effects of self-pollen tube inhibition. Therefore, the SIV-PGT system has broad prospects for SI research.

### Ovaries play an important role in SI occurrence in *C. oleifera*

Usually, self-pollinated pollen tubes in SSI plants, such as *Brassica oleracea* and *Arabidopsis lyrata*, cannot penetrate the stigma, and *SRK* and *SCR/SP11* play key roles in this process [47, 48]. Self-pollinated pollen tubes in GSI plants, such as *Malus domestica* and *Pyrus pyrifolia*, are arrested halfway up the style, and *S-RNase* and *SLF/SFB* play key roles in this process [9, 49]. Strong inhibition of self-pollen tubes occurs at the stigma or in the middle of the style, which is a typical feature of plants with SI. However, the inhibition of *C. oleifera* self-pollen tubes occurs near the ovary, which is the only cytological evidence thus far that the genus *Camellia* differs from other SI plants. The key factor involved in self-pollen tube inhibition in *C. oleifera* has not yet been identified, and whether it occurs strongly in the ovary needs to be investigated. In this study, we clarified the important role of the ovary in self-pollen tube inhibition by comparing SIV-PGT systems with and without inclusion of the ovary. The addition of the ovary to the SIV-PGT system specifically exacerbated the inhibition of the self-pollen tubes in SP, implying the importance of the ovary for SI in *C. oleifera*. It is worth noting that we emphasize the value of the ovary for *C. oleifera* SI and do not deny that the style also has an inhibitory effect on the self-pollen tubes of this species (Fig. 1B – d, e, and f). Studies have shown that the pollen tube growth rate of SP is slower than that of CP before the pollen tubes begin growing near the ovary [18]. Combined with the transcriptomic studies of CP and SP styles of *Pyrus* species at the beginning of pollination [50], we speculate that the plant SI response does not only occur during the period when the self-pollen tubes are most strongly inhibited, but also during the whole process of self-pollination from germination to growth cessation. This study validates the important contribution of the ovary to *C. oleifera* SI, which is a new interpretation, as well as its cytological characteristics *in vivo*.

### High levels of galloylated catechins are closely related to the SI of *C. oleifera*

The identification of *S*-factors, such as SRK, SCR/SP11, S-RNase, SLF/SFB, PrsS, and PrpS, often requires population materials containing genetic information on self-compatibility mutations, supported by map clones and transgenic evidence [51–58]. For *Camellia*, the lack of both self-compatible material and transgenic systems hinders the identification of SI determinants. Based on this, there are several multi-omics applications regarding the genus *Camellia*. Considering that the self-pollen tubes of *Camellia* plants can break through the stigma like those of GSI plants, some researchers have suggested that the cytotoxic theory of *S-RNase* genes also applies to self-pollen tube inhibition in *Camellia* species [33, 34]. However, some studies have found that *S-RNase* genes and their encoded proteins were not detected or were not significantly enriched in the omics data of self-pollinated samples [19, 20, 35]. Furthermore, gene expression profiles, structural composition, and chromosomal location based on *C. lanceoleosa* genomes suggest that the SI of *C. lanceoleosa* may favor a new mechanism distinct from *S-RNase*-based SI [27]. Therefore, more research is needed on the significance of *S-RNase* genes for SI in the genus *Camellia*. In addition, non-*S*-factors, such as hormones, ROS-related enzymes, and MAPK signaling substances, are also thought to be involved in SI [59–61]. The roles of these substances in the regulation of pollen tube growth have been widely proven; however, evidence for their direct involvement in SI is lacking. Secondary metabolites, such as flavonoids, are also believed to actively participate in the process of pollen tube growth in different plants [21–24], but there has been little research on whether they can specifically interfere with self-pollen tube inhibition to participate in SI [62].

**Figure 6:**
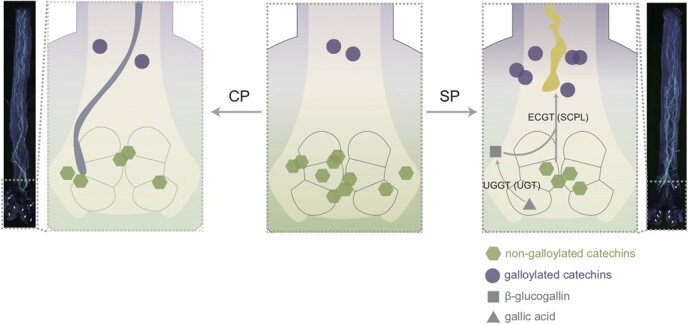
Model diagram of the inhibition of *Camellia oleifera* self-pollen tubes related to a high level of galloylated catechins. A darker background color indicates a higher relative content of non-galloylated catechins or galloylated catechins in the ovary, and vice versa (purple indicates galloylated catechins and green indicates non-galloylated catechins). Gray symbols indicate substances that were not detected in this project. The solid arrow indicates ovules and the dashed arrow indicates pollen tubes.

In this study, the newly constructed SIV-PGT system showed that with strong inhibition of SP self-pollen tubes, the relative content of galloylated catechins in the culture solution was significantly increased, while the CP pollen tubes maintained normal morphology and the level of galloylated catechins in the culture medium remained low. This is the first evidence linking galloylated catechins to pollination compatibility in *C. oleifera*. The *UGT* and *SCPL* families are the two key factors involved in the galloylation of non-galloylated catechins. Members of these families, either alone or in combination, convert non-galloylated catechins to galloylated catechins [31, 32]. In this study, several *UGT* and *SCPL* family genes and their encoded proteins were identified, and the enzymatic activities of both protein families were determined. The results showed that regardless of the gene transcription level, the abundance of the encoded proteins, or the enzyme activity, there were always several key members that exhibited higher levels in SP than in CP. This may be one of the important reasons why SP induced high levels of galloylated catechins in *C. oleifera*. Protein interaction network analysis showed that UGT and SCPL proteins may not act alone in the inhibition of self-pollen tubes in *C. oleifera*, but rather they require multiple components that coordinate with each other, including pollen tube growth-related proteins such as ABCF4, UBA2A, AHA6, OLE6, ADH1, ENODL1, BAHD, and GDSL [63–69]. Furthermore, studies have shown that amino acid polymorphisms of UGT89A2 (a member of the UGT protein family) affect the differential accumulation of dihydroxybenzoic acid (DHBA) glycosides in distinct *Arabidopsis* natural accessions [70]. It is worth investigating whether polymorphisms of *UGT* and *SCPL* (the members that specifically control galloylated catechin biosynthesis) in different *C. oleifera* varieties affect the differential accumulation of galloylated catechins and ultimately affect the degree of self-compatibility. Therefore, in *C. oleifera*, SP may induce the expression of *UGT* and *SCPL* genes and their encoded and interacting proteins, which then leads to elevated galloylated catechin levels and ultimately to SI (Fig. 6).

In addition, there may be specific SI signals in *C. oleifera* that induce the high expression of *UGT* and *SCPL*, as well as a high galloylated catechin content. Based on the enrichment analysis of DAPs in the proteome results, we found a significant enrichment of the glycosyl hydrolase (GH) family (Fig. 4B). Together with glycosyltransferase, GHs contribute to plant glycosylation, which has been shown to be involved in plant pollen tube recognition and successful fertilization [71, 72]. GHs are also involved in pollen tube growth [73]. In our study, based on the abundance of all GH family members in the proteome, we found that the abundance of most family members was higher in SP, and the abundance of more than three-fifths of the members was different among the three groups (Table S8, see online supplementary material). Therefore, GHs might also be involved in the recognition of *C. oleifera* self-pollen tubes and pistils. Protein interaction analysis showed that the absolute value of the correlation coefficient between GHs and SCP reached 0.92 (Fig. 4C). Thus, we speculated that the enriched GHs in *C. oleifera* may play an important role in the high content of galloylated catechins in SP. However, this speculation requires further in-depth verification.

In conclusion, we created a new SIV-PGT system that can avoid the interference of the genetic background to more accurately search for substances that inhibit the growth of *C. oleifera* self-pollen tubes. Using the SIV-PGT system, we verified the importance of ovary for SI in *C. oleifera*. In addition, we propose for the first time that high levels of galloylated catechins play an important role in the inhibition of self-pollen tubes, which is of great significance for the in-depth study of the mechanism of SI in *C. oleifera*. The results of this study provide new ideas regarding the SI of *C. oleifera* and help to expand the functional research on catechins in plant sexual reproduction.

## Materials and methods

### Plant materials and pollination designs

The plant materials were five-year-old grafted seedlings planted in a *C. oleifera* planting base in Liuyang, Hunan, China. The rootstocks of the grafted seedlings were *C. oleifera*, and the scions were cultivars ‘Huashuo’ (HS) and ‘Huaxin’ (HX). The anthers of ‘HS’ and ‘HX’ were harvested when the buds were enlarged but not expanded, incubated at 25°C until the pollen was dispersed, and then stored at −20°C. The strategy of ‘HX’ × ‘HS’ (cross-pollination, CP) was to manually apply the pollen of ‘HS’ to the stigma of emasculated ‘HX’, and the strategy of ‘HX’ × ‘HX’ (self-pollination, SP) was to artificially smear the pollen of ‘HX’ onto its own stigmas. The pistils of unpollinated (UP) ‘HX’ served as a control. Pollination was followed by bagging to prevent crosstalk of the pollen. The test was conducted between 9 a.m. and 11 a.m. on sunny days between October and December 2021.

### Semi-*in vivo* pollen tube growth test (SIV-PGT) system

The test used for multi-omics sampling was based on the method described by Hafidh *et al.* for SIV-PS, with some modifications [39, 40]. The procedure was as follows: 24 h after pollination (HAP), CP and SP flowers were harvested and brought back to the laboratory. The receptacles were quickly cut off and then cut along the junction of the styles and ovaries. The ovaries were then cut longitudinally in half. The styles and halved ovaries were assembled separately into the SIV-PGT devices, which were prepared before the flowers were picked (each device contained eight pistils, and the culture solution consisted of 10% sucrose, 0.01% boric acid, and pure water [w/v]), taking care that the style incision extended below the surface of the culture solution. The assembled SIV-PGT devices were then placed in an incubator at 23–25°C with soaked but non-dripping cotton wool placed inside to provide humidity, and were incubated for 24 h. Phenotypes of the CP and SP pollen tubes protruding from the respective style incisions in the culture medium were observed.

### Phenotypic analysis of pollen tubes

Semi-*in vivo*: At 48 HAP, the styles were pulled out of the SIV-PGT devices with forceps and placed on a slide. A drop of 0.2% (w/v) aniline blue solution containing 1 × PBS was applied to the style incision, and the phenotypes of the pollen tubes were observed microscopically (Olympus BX-51, Japan) and recorded. *In vitro*: The pollen of ‘HS’ and ‘HX’ was evenly sprinkled on the solid medium [10% sucrose, 0.01% boric acid, 1% agar, and pure water (w/v)]. Then, the Petri dishes were sealed and placed in an incubator at 23–25°C for 48 h, after which the pollen tubes were photographed for phenotyping and statistical analysis. *In vivo*: Pistils of CP and SP at 48 HAP were fixed with 25% acetic acid in ethanol, hydrated with an ethanol series (70%, 50%, and 30% ethanol), and dissected such that the placenta unfolded along the ventral suture with the corresponding style to expose the complete growth trajectory of the pollen tubes growing in the pistil from the stigma to the ovary. This was followed by treatment with 8 M NaOH for 2 h to allow softening. To observe the penetration of the pollen tubes into the micropyle, the pistil was soaked in a solution of NaClO with an effective chlorine content of 9000 mg·L^−1^ for 2 h before softening. The pollen tube phenotypes in each pistil were observed using a fluorescence microscope after staining with a 0.2% (w/v) aniline blue solution.

### Analysis of the metabolites and proteins present in the culture solution of the SIV-PGT system

At 48 HAP, the culture solution in each SIV-PGT device was filtered into centrifuge tubes, snap-frozen in liquid nitrogen, and stored at −80°C. Half of the samples were taken for metabolomic and proteomic analyses, and the sample volumes of UP, CP, and SP culture solutions for both the metabolome and proteome were approximately 200 mL × 3 biological replicates. Freeze-dried samples for metabolomic analysis were analysed using a UPLC-ESI-MS/MS system after substance extraction. The substance was qualitatively based on the secondary spectrum information of the MWDB database [74]. The multiple reaction monitoring modes of the triple quadrupole mass spectrometer were used to combine the area of the mass spectrum peak for substance quantification. Sampling and pooling of all samples for quality control (QC) and the total ion chromatogram map of the mass spectrometry analysis of the QC samples were used to demonstrate the repeatability of metabolite extraction and detection. DAMs were determined by VIP ≥ 0.8 and absolute fold change (FC) ≥ 1.5.

Samples for proteomic analysis were concentrated by ultrafiltration, followed by protein extraction and enzymatic digestion. The peptides were separated using the EASY-nLC 1200 UHPLC system and analysed using an Orbitrap Exploris™ 480 mass spectrometer. The data-acquisition mode used a data-dependent scanning program. The resulting MS/MS data were processed using Proteome Discoverer (v2.4.1.15). Tandem mass spectra were searched against the protein sequence database of the *C. oleifera* transcriptome (58 031 sequences). Data quality was assessed using the peptide length distribution. The relative quantitative value (R) of proteins in different samples was calculated using the formula *R_ij_* = *I_ij_* / Mean(*I_j_*) (‘I’, intensity after mean centering; ‘i’, samples; and ‘j’, proteins). DAPs were determined by *P* < 0.05 and absolute FC ≥ 1.5. Detailed methods for the detection and enrichment analysis of DAMs and DAPs are included in Appendix S1 (see online supplementary material).

### Catechin treatment

To explore the effects of different catechins on pollen tube growth, dissolved EC, ECG, and EGCG (Solarbio, Beijing, China) were added to a pollen tube growth medium *in vitro* at final concentrations of 1, 5, 25, 125, and 625 μg·mL^−1^. Treatment concentrations (50, 100, 200, and 400 μg·mL^−1^) were then optimized to highlight the differences in the effects of non-galloylated catechins and galloylated catechins on pollen tube growth *in vitro*. To further verify the effect of high levels of galloylated catechins on the SI of *C. oleifera*, the medium in the SIV-PGT system was modified to contain 100 μg·mL^−1^ of EC, ECG, or EGCG in the solid form. The CP and SP styles and the corresponding halved ovaries were laid flat on different kinds of solid medium (each 6-mm Petri dish contained 1 pistil). At 48 HAP, phenotypic differences between the CP and SP pollen tubes protruding from the style incision were observed and recorded.

### Multi-omics association analysis

A dynamic network heatmap was used to show the correlation between the 45 DAPs screened from the results of domain enrichment-based clustering and their respective correlations with seven catechins in the metabolome. The Pearson correlation coefficient was used to characterize the degree of correlation, and the threshold was set as *P* < 0.05 and |*r* | ≥ 0.8. A total of 135 *UGT* and 49 *SCPL* family genes were screened from the Unigenes library using blastp, with the relevant *Camellia sinensis* sequence as the query [32, 75] and CD-search. The FPKM of these genes was correlated with the contents of various catechins, and the results (*P* < 0.05 and |*r*| ≥ 0.8) were displayed in a cluster heat map to highlight the possible involvement of galloylated catechin biosynthesis family gene members. Quantitative information on all the quantifiable proteins was also used to calculate the Pearson correlation coefficients and *P*-values, and then the cytoHubba plugin of Cytoscape3.8.2 was used to extract and visualize the proteins interacting with UGT and SCPL.

### Statistical analysis

The sample sizes used to calculate pollen tube length varied from experiment to experiment. Specifically, they were: 3 (culture devices) × 6 (biological replicates) for semi-*in vivo* experiments; 2 (Petri dishes) × 3 (biological replicates) for *in vitro* experiments; and 8 (pistils) × 3 (biological replicates) for *in vivo* experiments. Pollen tube lengths were counted using ImageJ, and an analysis of the significant differences was performed using SPSS26. The *t*-test was used for all analyses, except that for the catechin treatment, which used ANOVA.

Box plots and column charts were created using Microsoft Excel. Data are expressed as the mean ± standard error. ‘n’ denotes the number of pollen tubes. Asterisks directly above the data bars indicate a significant difference (* or ** = *P*-value <0.05 or 0.01) and ‘n.s.’ means no significant difference. Letters indicate significantly different fractions (*P* < 0.05). The statistical information used in this study is shown in the legends of the corresponding figures.

## Acknowledgement

Our work was supported by the National Key R&D Program of China (2018YFD1000603-1), the Natural Science Foundation of Hunan Province (2020JJ5968), Scientific Research Foundation for Advanced Talents of Central South University of Forestry and Technology (2018YJ002), Special Funds for the Construction of Innovative Provinces in Hunan (2021NK1007) and the Key Program of Education Department of Hunan Province (grant no. 20A524). We thank Dr. Yajun Liu for assistance with the galloylated catechin biosynthesis analysis.

## Author contributions

Y.C., W.G., and D.Y. designed the experiment; Y.C., J.X., and H.G. carried out the experiment; Q.S. participated in the multi-omics data analysis; Y.C. wrote the manuscript; W.G. and S.X revised the manuscript. All authors read and approved the final manuscript.

## Data availability

Metabolomics data have been deposited to the EMBL-EBI MetaboLights database with the identifier MTBLS5346. The mass spectrometry proteomics data have been deposited to the proteomeXchange Consortium via the PRIDE partner repository with the dataset identifier PXD035406.

## Conflict of interest

The authors declare that they have no conflicts of interest associated with this work.

## Supplementary data

Supplementary data is available at *Horticulture Research Journal* online.

## Supplementary Material

Web_Material_uhac248
