# Supplementary material for: Integration of semi-in vivo assays and multi-omics data reveals the effect of galloylated catechins on self-pollen tube inhibition in Camellia oleifera
Source: Hortic Res. 2022 Nov 10;10(1):uhac248. doi: 10.1093/hr/uhac248 (PMC9832949; doi:10.1093/hr/uhac248)
Supplement: Web_Material_uhac248 [file web_material_uhac248.zip › Supplementary data 1.docx]

Integration of semi-*in vivo* assays and multi-omics data reveals the effect of galloylated catechins on self-pollen tube inhibition in *Camellia oleifera*

Yihong Chang; Wenfang Gong; Jinming Xu; Han Gong; Qiling Song; Shixin Xiao; Deyi Yuan

**Supplementary data 1**

**Figure S1–6 and Appendix 1**

**Fig. S1 Morphology of pollen tube growth *in vitro*.** Scale bars, 1 mm.

**
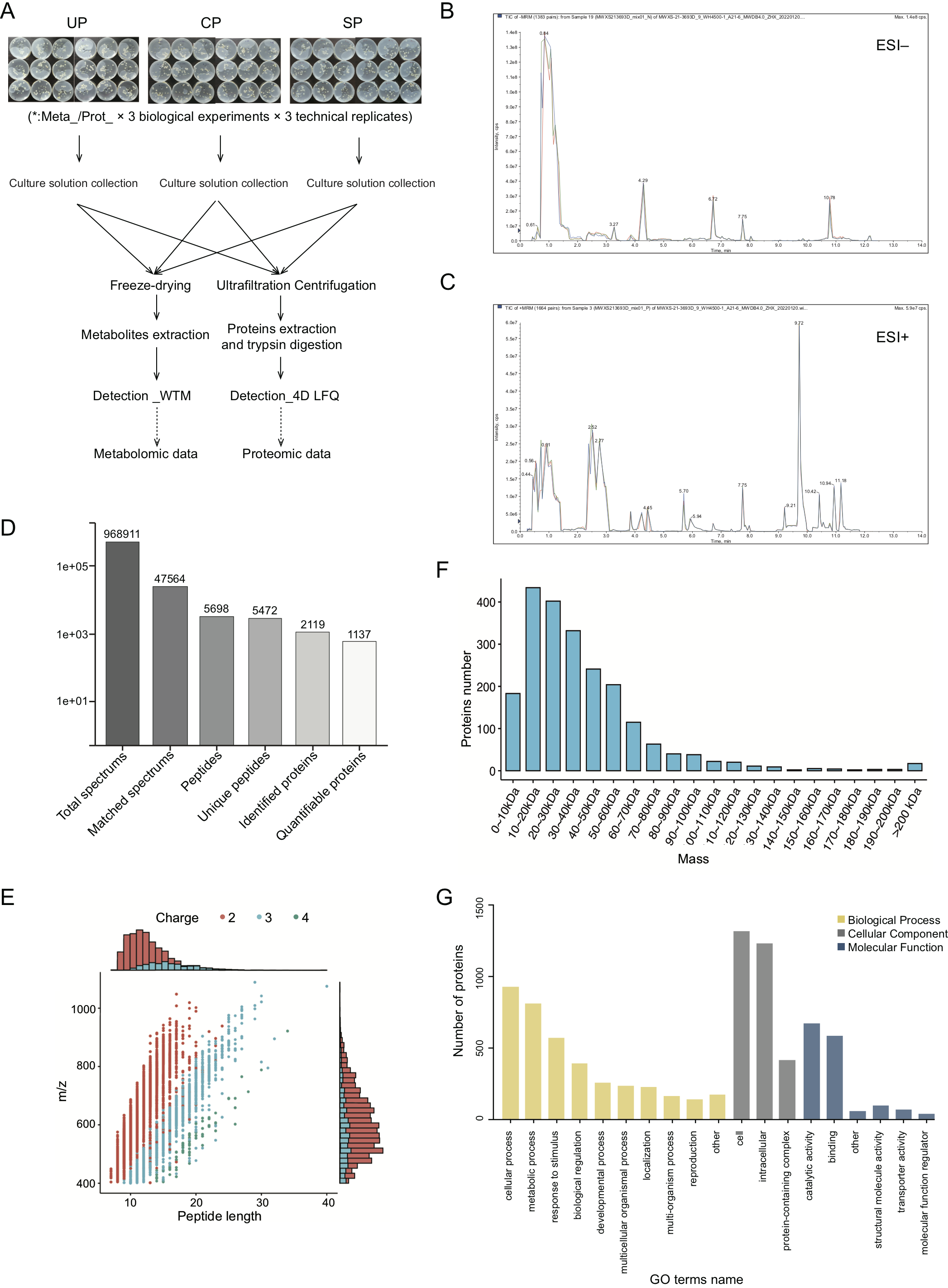
**

**Fig. S2 Basic information of detected metabolites and proteins.** (A) Flow chart of SIV-PGT system operation and sampling. The strategies for quantifying the detected metabolites and proteins were Widely Targeted Metabolome (WTM) and 4D Label-free Quantitative Proteomics Analysis (4D LFQ). (B) and (C) represent the overlay of total ion chromatograms (TIC plots) detected by mass spectrometry of QC samples. (D) Number of the identified peptides and proteins after data filtering in the library search results. (E) and (F) represent the distributions of peptide length and protein molecular weight, respectively. (G) Functional classification of the identified proteins in the Gene Ontology (GO) database.

**
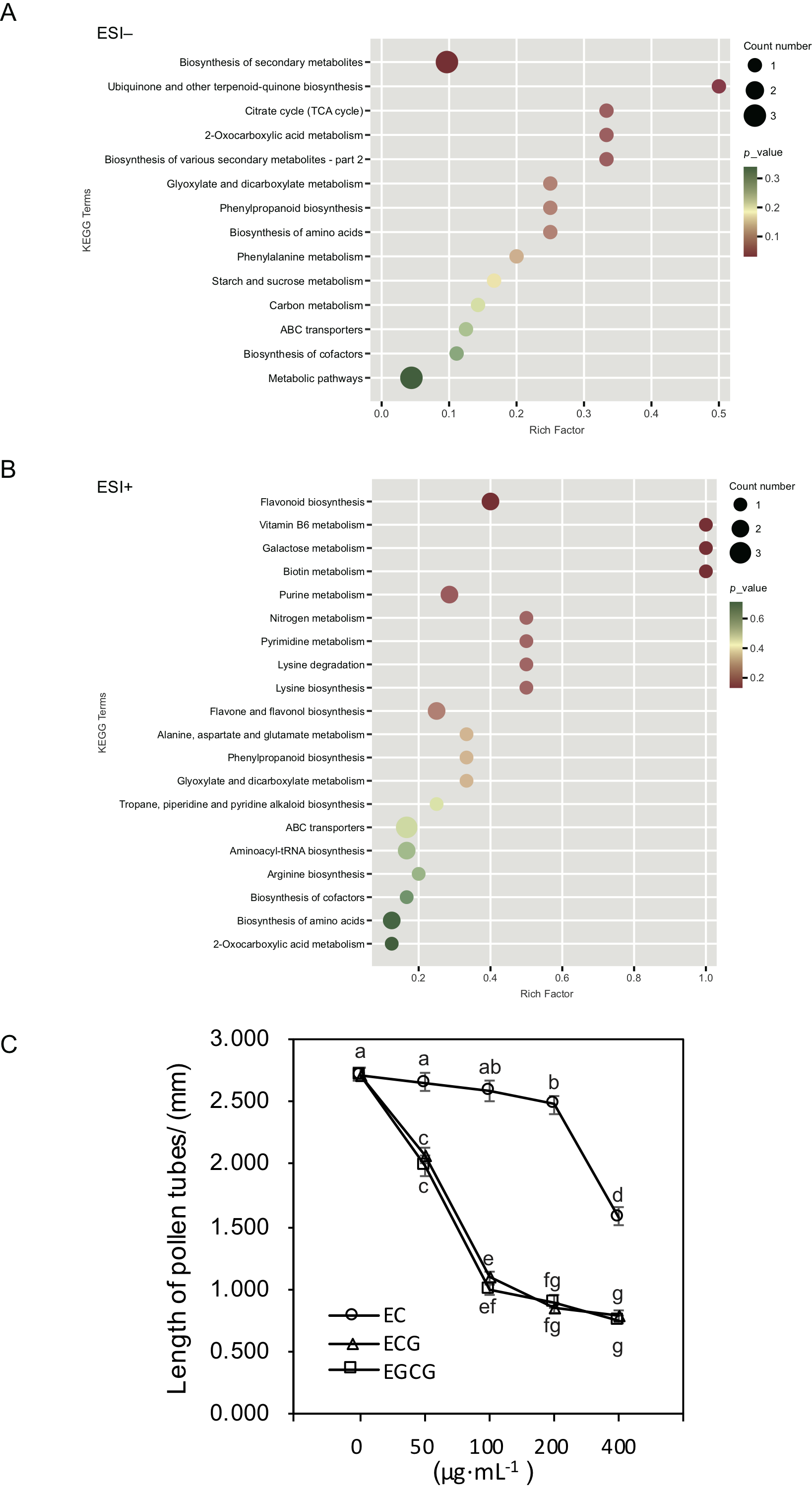
**

**Fig. S3 KEGG enrichment bubble plot of 38 DAMs (CP_vs_SP).** The analysis was performed using ESI− (A) or ESI+ (B). (C) Broken line diagram of the length of *C. oleifera* pollen tubes grown *in vitro* under different concentrations of catechins. Letters indicate significantly different fractions (ANOVA, *p* < 0.05). n ≥ 35.

**Fig. S4 KEGG functional classification (A) and enrichment (B) of 36 DAPs (CP_vs_SP).**

**
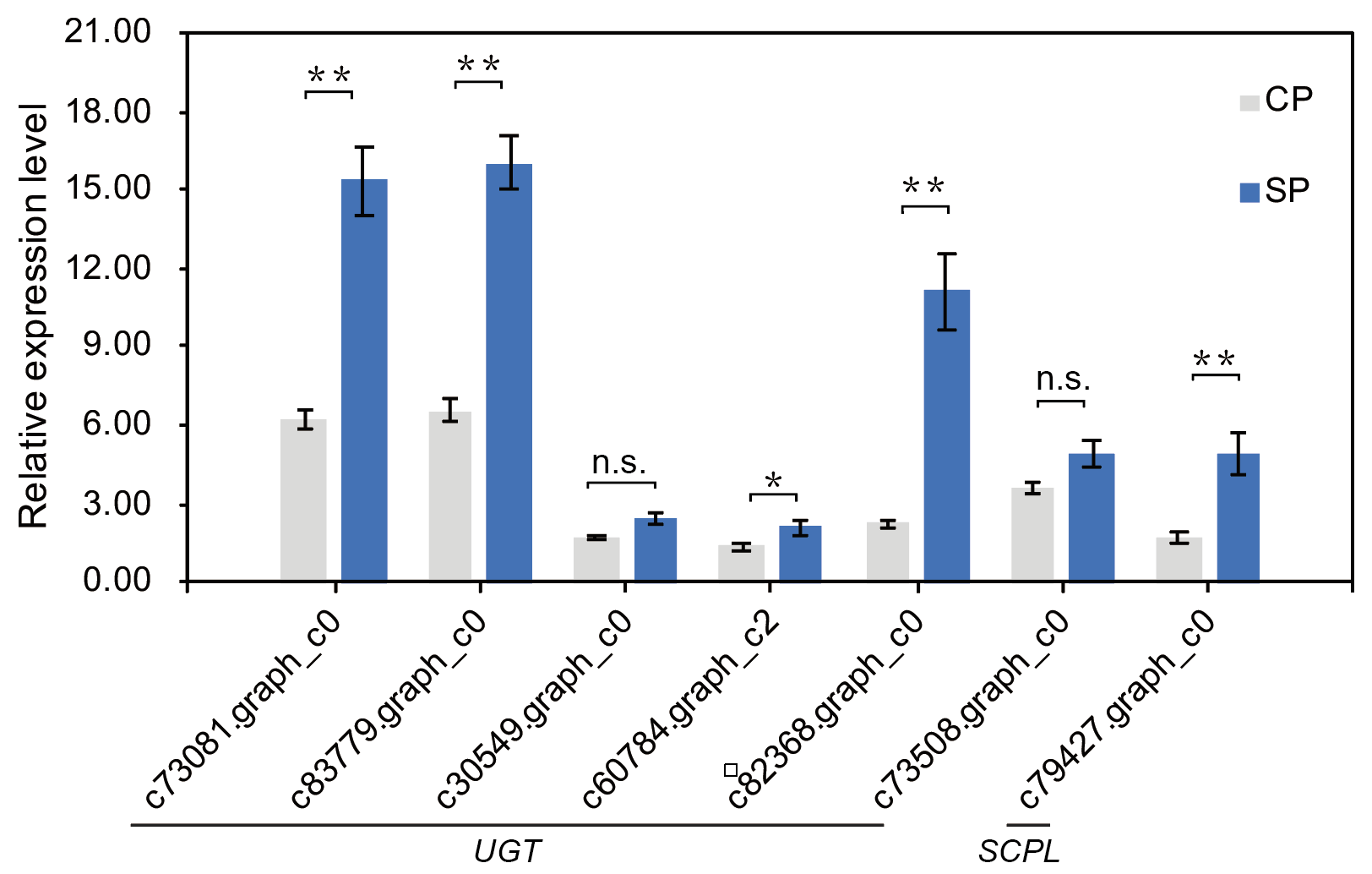
**

**Fig. S5 Expression of candidate *UGT* and *SCPL* genes in CP and SP pistils at 48 HAP.** Error bars indicate the standard error (SE) of three biological replicates (*t*-test, **p* < 0.05, ***p* < 0.01, “n.s.” means no significant difference).


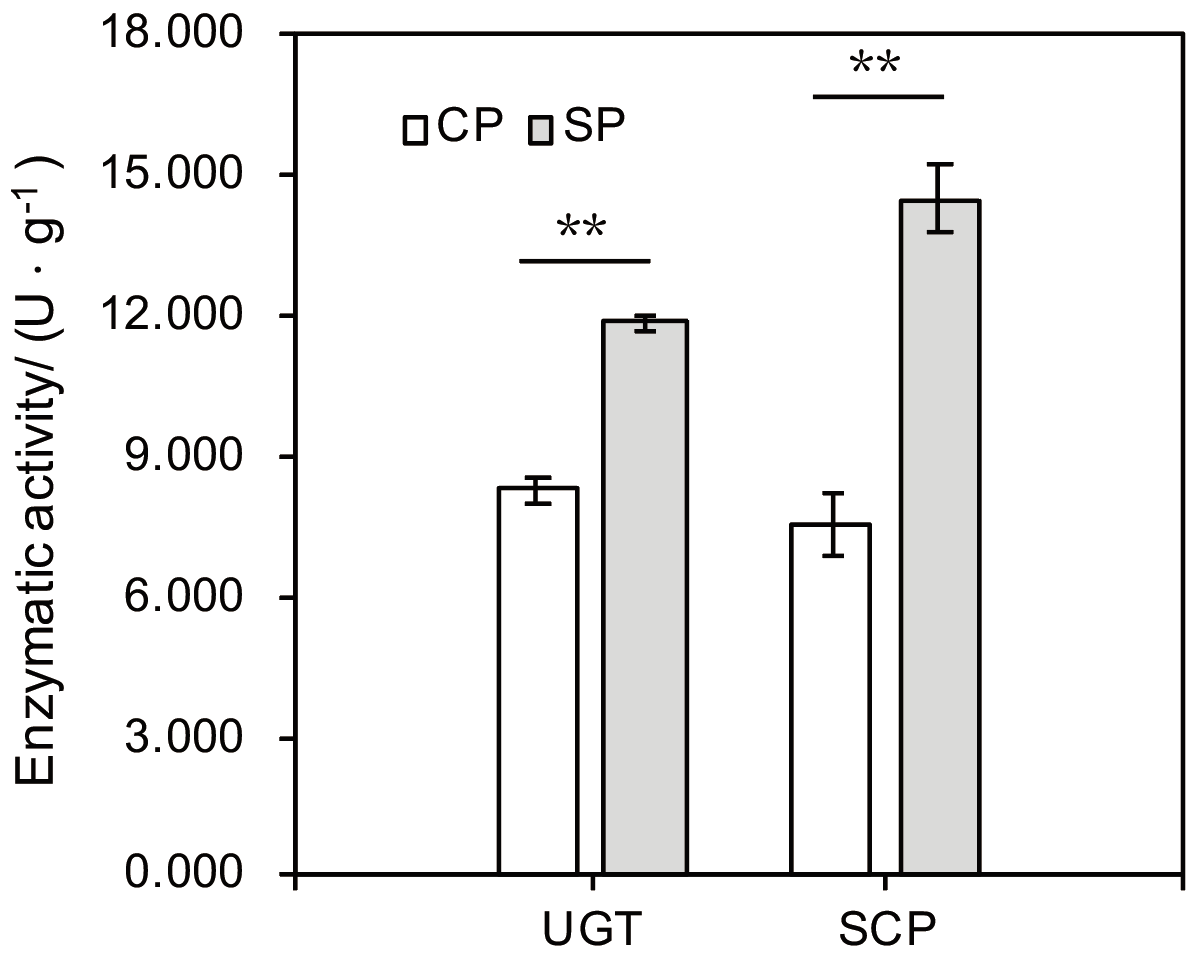


**Fig. S6 Activities of UGT and SCP protein family enzymes in CP and SP pistils at 48 HAP.** Error bars indicate the standard error (SE) of three biological replicates (*t*-test, **p* < 0.05, ***p* < 0.01).

**Appendix 1** Detailed methods for metabolite and protein detection and analysis, transcriptome sequencing, qRT-PCR, and ELISA measurements.

**Metabolites extraction**

(1) Put the samples of culture solution into a freeze dryer (Scientz-100F) for vacuum freeze drying; (2) Grind (30 Hz, 1.5 min) with a grinder (MM 400, Retsch) to powder; (3) Weigh 100 mg of powder and dissolve it in 1.2 mL of 70% methanol extract; (4) Vortex every 30 minutes for 30 seconds for a total of 6 vortexes, and place the samples in a 4°C refrigerator overnight; (5) After centrifugation (12,000 rpm, 10 minutes), aspirate the supernatant, filter the sample with a 0.22-μm microporous membrane (SCAA-104, ANPEL, China), and store it in a sample vial for UPLC-MS/MS analysis.

**Chromatographic mass spectrometry acquisition conditions**

The data acquisition instrument system mainly included Ultra Performance Liquid Chromatography (UPLC) (SHIMADZU Nexera X2, https://www.shimadzu.com.cn/ ) and Tandem mass spectrometry (MS/MS) ( Applied Biosystems 4500 QTRAP, http://www.appliedbiosystems.com.cn/).

   Liquid phase conditions mainly included: (1) Column: Agilent SB-C18 1.8-µm, 2.1 mm × 100 mm; (2) Mobile phase: phase A was ultrapure water (add 0.1% formic acid), phase B was acetonitrile (add 0.1% formic acid); (3) Elution gradient: The ratio of phase B was 5% at 0.00 min, the ratio of phase B increased linearly to 95% within 9.00 min, and was maintained at 95% for 1 min; 10.00~11.10 min, the ratio of phase B decreased to 5%, and equilibrated at 5% to 14 min; (4) Flow rate 0.35 mL ∙ min^-1^; column temperature 40°C; injection volume 4 μL.

   The mass spectrometry conditions mainly included: LIT and triple quadrupole (QQQ) scans were acquired on a triple quadrupole linear ion trap mass spectrometer (Q TRAP), AB4500 Q TRAP UPLC/MS/MS system equipped with an ESI Turbo ion spray interface that can be Analyst 1.6.3 software (AB Sciex) controlled to run both positive and negative ion modes. ESI source operating parameters were as follows: ion source, turbo spray; source temperature 550°C; ion spray voltage (IS) 5500 V (positive ion mode)/–4500 V (negative ion mode); ion source gas I (GSI), gas II (GSII) and curtain gas (CUR) was set to 50, 60 and 25.0 psi, respectively, and the collision-induced ionization parameter was set to high. Instrument tuning and mass calibration were performed with 10 and 100 μmol ⋅ L^-1^ polypropylene glycol solutions in QQQ and LIT modes, respectively. QQQ scans used MRM mode and set the collision gas (nitrogen) to medium. Through further DP and CE optimization, the DP and CE of individual MRM transitions were completed. A specific set of MRM transitions was monitored in each epoch based on the metabolites eluted in each epoch.

**Differential abundance metabolites Screening**

VIP values were extracted from the OPLS-DA result, which was generated using the R package MetaboAnalystR. The data was log transform (log_2_) and mean centering before OPLS-DA. In order to avoid overfitting, a permutation test (200 permutations) was performed.

**Protein extraction and enzymatic digestion**

All samples of culture solution were centrifuged at 12,000 g for 10 min at 4°C to remove solid impurities. The supernatant was then transferred to an ultrafiltration tube (Millipore) and concentrated to 0.5 mL by centrifugation at 5,000 g at 4°C. The buffer was exchanged once with 8 M urea (containing 1% protease inhibitors), and the protein concentration was determined using the BCA kit. Equal amounts of each sample protein were digested, adjusted to the same volume with lysis solution, then dithiothreitol (DTT) was added to a final concentration of 5 mM and reduced for 30 min at 56°C. Iodoacetamide (IAA) was added to a final concentration of 11 mM and incubated for 15 min at room temperature and protected from light. The alkylated sample was transferred to an ultrafiltration tube, centrifuged at 12,000 g for 20 min at room temperature, exchanged 3 times with 8 M urea, then replaced urea 3 times with exchange buffer, added trypsin at a ratio of 1:50 (protease: protein, m/m) and digested overnight. The peptides were recovered by centrifugation at 12,000 g for 10 min at room temperature, then recovered once with ultrapure water and the two peptide solutions were combined.

**UPLC-MS/MS analysis**

The peptides were dissolved in phase A of the liquid chromatography mobile phase and separated using EASY-nLC 1200 ultra-high performance liquid phase system. Mobile phase A was an aqueous solution containing 0.1% formic acid and 2% acetonitrile; mobile phase B was an aqueous solution containing 0.1% formic acid and 90% acetonitrile. Liquid gradient settings: 0–68 min, 6–23% B; 68–82 min, 23–32% B; 82–86 min, 32–80% B; 86–90 min, 80% B, The flow rate was maintained at 500 nL ∙ min^-1^. Peptides were separated by a UHPLC system and injected into an NSI ion source for ionization before entering the Orbitrap Exploris™ 480 mass spectrometer for analysis. The ion source voltage was set to 2.3 kV, the FAIMS compensation voltage (CV) was set to –45V, –65V, and the peptide precursor ions and their secondary fragments were detected and analyzed using high-resolution Orbitrap. The scanning range of the primary mass spectrometer was set to 400~1200 m/z, and the scanning resolution was set to 60000; the fixed starting point of the scanning range of the secondary mass spectrometer was 110 m/z, and the secondary scanning resolution was set to 15000, and the TurboTMT was set to Off. The data acquisition mode used a data-dependent scanning (DDA) procedure, that was, after the first-level scan, the top 25 peptide precursor ions with the highest signal intensity were selected and sequentially entered into the HCD collision cell for fragmentation using 27% of the fragmentation energy. Secondary mass spectrometry analysis was also performed sequentially. In order to improve the effective utilization of mass spectrometry, the automatic gain control (AGC) was set to 100%, the signal threshold was set to 5E4 ions/s, the maximum injection time was set to Auto, and the dynamic exclusion time of tandem mass spectrometry scanning was set to 20 s to avoid repeated scans of precursor ions.

**Database search**

The secondary mass spectrometry data of this experiment were retrieved using Proteome Discoverer (v2.4.1.15). The protein sequence database was the result of the transcriptome of *C. oleifera* (5,8031 sequences), which added an anti-library to calculate the false positive rate (FDR) caused by random matches, and added a common contamination library to it to eliminate the influence of contaminating proteins in the identification results; the digestion method was set to Trypsin (Full); the number of missed cleavage sites was set to 2; the minimum length of the peptide was set to 6 amino acid residues; the maximum number of peptide modifications was set to 3; the mass error tolerance of primary precursor ions was set to 10 ppm, and the mass error tolerance of secondary fragment ions was 0.02 Da. Carbamidomethyl (C) was set as fixed modification, Oxidation (M), Acetyl (N-terminus), Met-loss (M), Met-loss+acetyl (M) were set as variable modification. Proteins, peptides, and FDRs identified by PSM were all set to 1%. In order to obtain high-quality analysis results, further data filtering was required for the search database analysis results. The accuracy FDR of identification at the three levels of the spectrum, peptide, and protein was set to 1%; the identification of protein needed to contain at least one unique peptide.

**Transcriptome analysis of CP and SP pistils in *C. oleifera***

At 48 HAP, the pistils of CP, SP, and UP were taken and temporarily stored at −80°C after being quick-frozen in liquid nitrogen. The samples size of UP, CP, and SP pistils used for the transcriptome respectively, were 8 × 3 biological replicates. RNA was extracted from pistil samples using a Total RNA Extraction Kit (Omega, Norcross, GA, USA). After the cDNA library was constructed and tested, the library preparations were sequenced on an Illumina Hiseq 2000 platform and paired-end reads were generated. After raw data was filtered, the clean data was sequence assembled using Trinity software to obtain the Unigene library of the species. The Fragments Per Kilobase of transcript per Million mapped reads（FPKM）value was used to indicate the expression abundance of the corresponding Unigene. Differential expression genes (DEGs) were determined by the corrected *p*-value, False Discovery Rate (FDR) < 0.05, and absolute FC ≥ 1.5.

**Functional enrichment analysis**

Identified metabolites were annotated using the KEGG compound database, and annotated metabolites were then mapped to the KEGG pathway database. Pathways with significantly regulated metabolites mapped to were then fed into metabolite sets enrichment analysis (MSEA), their significance was determined by the hypergeometric test’s *p*-values. KEGG database was also used to identify enriched pathways by a two-tailed Fisher’s exact test to analyze the enrichment of the DAPs against all identified proteins. The pathway with a corrected *p*-value < 0.05 was considered significant. These pathways were classified into hierarchical categories according to the KEGG website.

For each category protein, the InterPro database was researched and a two-tailed Fisher’s exact test was employed to analyze the enrichment of the DAPs against all identified proteins. Protein domains with a corrected *p*-value < 0.05 were considered significant. For further hierarchical clustering based on DAPs domains classification, we first collated all the categories obtained after enrichment along with *p*-values of domain enrichment and then filtered for those categories which were at least enriched in one of the clusters with a *p*-value < 0.05. This filtered *p*-value matrix was transformed by the function x=−log_10_ (*p*-value). Finally, these x values were z-transformed for each functional category. These z scores were then clustered by one-way hierarchical clustering (Euclidean distance, average linkage clustering) in Genesis.

**Quantitative real-time PCR (qRT-PCR) analysis**

Pistils of CP and SP at 48 HAP were used for qRT-PCR analysis. The specific primers of 6 *UGT* and 1 *SCPL* genes were designed with Primer Premier 5.0, and *EF1α* was used as the reference gene (Table S9). The Evo M-MLV RT Kit with gDNA Clean for qPCR kit (Accurate Biology, China) was used to synthesize first-strand cDNA following the manufacturer’s specifications. The qRT-PCR was performed using the SYBR® Green Premix Pro-Taq HS qPCR Kit (Accurate Biology, China) as described in the manufacturer’s instructions. The relative expression levels of 7 genes were calculated according to the 2^–ΔCt^ method with three biological duplications. Data are expressed as mean ± standard error. Asterisks directly above the data bars indicate a significant difference (*t*-test, **p*-value < 0.05, ***p*-value < 0.01, “n.s.” means no significant difference).

**ELISA measurement**

Pistils of CP and SP at 48 HAP were used for ELISA analysis. UDP-glycosyltransferase (UGT) and serine carboxypeptidase (SCP) were, respectively, quantified using Plant UDP-glucose Glycoprotein Glucosyltransferase ELISA Kit no. KT0538-B (MAISHA INDUSTRIES, China) and Plant Serine Carboxypeptidase ELISA Kit no. KT0531-B (MAISHA INDUSTRIES, China). Assays were performed according to the manufacturer's instructions. A standard curve was generated and used to determine UGT and SCP activity in samples. A total of 8 pistils were mixed to determine the enzyme activities of UGT and SCP, respectively. Each experiment was repeated three times with consistent results. Significant differences between CP and SP were calculated with SPSS26 (*t*-test).
